# Transcriptomic Analyses of Myeloid-Derived Suppressor Cell Subsets in the Circulation of Colorectal Cancer Patients

**DOI:** 10.3389/fonc.2020.01530

**Published:** 2020-09-02

**Authors:** Varun Sasidharan Nair, Reem Saleh, Salman M. Toor, Nehad M. Alajez, Eyad Elkord

**Affiliations:** Cancer Research Center, Qatar Biomedical Research Institute (QBRI), Hamad Bin Khalifa University (HBKU), Qatar Foundation (QF), Doha, Qatar

**Keywords:** colorectal cancer, RNASeq, myeloid-derived suppressor cells, JAK/STAT, histone acetylation

## Abstract

Myeloid-derived suppressor cells (MDSCs) promote tumor immune evasion and favor tumorigenesis by activating various tumor-promoting downstream signals. MDSC expansion is evident in the circulation and tumor microenvironment of many solid tumors including colorectal cancer (CRC). We have recently reported the transcriptomic profiles of tumor-infiltrating MDSCs in CRC patients and uncovered pathways, which could potentially assist tumor progression. In this study, we sorted different subsets of circulating MDSCs in CRC patients and investigated their transcriptomic profiles in order to disclose pathways, which could potentially contribute to disease progression. The sorted subsets included polymorphonuclear/granulocytic MDSCs (PMN-MDSCs), immature MDSCs (I-MDSCs), and monocytic MDSCs (M-MDSCs). Our functional annotation analyses revealed that multiple pathways including DNA damage-, chemotaxis-, apoptosis-, mitogen-activated protein kinase-, transforming growth factor β-, and myeloid differentiation–related transcripts were higher in PMN-MDSCs, compared with monocytic antigen-presenting cells (APCs) or I-MDSCs. Furthermore, genes related to Janus kinase (JAK)–signal transducer and activator of transcription (STAT) were also elevated in PMN-MDSCs. These data suggest that upregulation of JAK-STAT pathway could trigger multiple downstream targets in PMN-MDSCs, which favor tumor progression. Additionally, we found that pathways including phosphatidyl inositol 3-kinase (PI3K), interleukin 6, and TGF-β in M-MDSCs and cell cycle–related pathways in I-MDSCs were upregulated, compared with monocytic APCs. Moreover, acetylation-related genes were upregulated in both PMN-MDSCs and M-MDSCs. This latter finding implicates that epigenetic modifications could also play a role in the regulation of multiple tumor-promoting genes in PMN-MDSCs and M-MDSCs. Taken together, this study reveals various signaling pathways, which regulate the function of MDSC subsets in the circulation of CRC patients. However, functional studies are warranted to support these findings.

## Introduction

Colorectal cancer (CRC) ranks second in terms of mortality and third in terms of incidence among all cancers in 2018 (1). Immune cells play an indispensable role in tumorigenesis and progression of CRC (2, 3). The infiltration of CD3^+^CD8^+^ T cells into the CRC tumor microenvironment (TME) has been reported as an indicator of disease prognosis; higher CD3^+^CD8^+^ T-cell infiltrates have been associated with favorable prognosis (2). Apart from lymphoid cells, the involvement of myeloid cells in the enhancement of metastatic cascade in solid tumors has also been reported (4). Myeloid-derived suppressor cells (MDSCs) play a predominant role in the survival of tumor cells by providing an immunosuppressive shield to protect them from host immune response and facilitate resistance to immunotherapy (4). It has been shown that MDSCs favor the survival of regulatory T cells within the TME, which are a key component of the immunosuppressive mediators that attenuate functionality of tumor-reactive T cells (5) and also induce the differentiation of fibroblasts to cancer-associated fibroblasts (6). Increase in circulating MDSC in higher stages of cancer has been reported to be correlated with worse survival rates and resistance to immune checkpoint blockade (7–9). These reports suggest that targeting MDSCs improves the host-immune responses against malignant cells and also enhances the efficiency of immune checkpoint blockade therapy.

Different MDSC subsets can suppress antitumor immune responses via distinct mechanisms (10). Monocytic MDSCs (M-MDSCs) can inhibit T-cell activation via arginase 1 (ARG1)–, inducible nitric oxide synthase–, and transforming growth factor β (TGF-β)–mediated signaling pathways (11, 12). In contrast, polymorphonuclear/granulocytic MDSCs (PMN-MDSCs), which are usually the most predominant MDSC subset in most cancers, can release high levels of reactive oxygen species and inhibit T-cell stimulation/activation in an antigen-specific manner (13–15). The presence of immature MDSCs (I-MDSCs) in tumor issues and peripheral blood of cancer patients and their relationship with poor prognosis has been reported (16); however, distinct immunosuppression-mediated mechanisms for I-MDSCs have not been yet elucidated.

We have previously reported that PMN-MDSCs and I-MDSCs were expanded in the CRC TME, whereas only PMN-MDSCs were expanded in circulation of CRC patients (3). Importantly, we found that the levels of circulating PMN-MDSCs correlated with tumor stage and histological grade in CRC patients (3). In this study, we investigated the transcriptomic analyses to reveal signaling pathways and biological mechanisms regulated by MDSC subsets in the circulation of CRC patients. We performed comparative analyses of the transcriptomic profiles of the different myeloid subsets, compared to monocytic antigen-presenting cells (APCs).

We found that multiple cancer-related pathways were upregulated in the different myeloid cell subsets. Interestingly, we found that acetylation-related genes were upregulated in both M-MDSCs and PMN-MDSCs, which could play a role in the transcriptional regulation of genes favoring tumor progression and metastasis of malignant cells. Collectively, our data reveal that tumor-promoting signaling pathways were upregulated in circulating myeloid suppressive cell subsets of CRC patients, suggesting their contribution to carcinogenesis and tumor progression. However, these data lack functional studies due to limited cell numbers following FACS sorting, and further confirmation studies are needed in a larger number of patients.

## Materials and Methods

### Sample Collection and Storage

Peripheral blood samples were obtained from 4 treatment-naive CRC patients (No. 7, 9, 12, and 16) at Hamad Medical Corporation, Doha, Qatar. Table 1 shows the clinical and pathological characteristics of participating patients. All patients provided written informed consent prior to sample collection. Peripheral blood mononuclear cells (PBMCs) were isolated from fresh blood using Histopaque gradient centrifugation and stored, as previously described (17). This study was performed under ethics approvals from Hamad Medical Corporation, Doha, Qatar (protocol no. MRC-02-18-012), and Qatar Biomedical Research Institute, Doha, Qatar (protocol no. 2018-018). All experiments were executed in accordance with relevant guidelines and regulations.

**Table 1:** Characteristic features of study populations.

	**CRC patients**
Number	4
Age	73 (48–96)^†^
Gender (male:female)	3:1
TNM stage	
IIA	3
IV	1 (patient 9)
Histological grade	
G2–moderately differentiated	All samples

† *Data shown represent median (range)*.

*CRC, colorectal cancer*.

### Multiparametric Flow Cytometry

Isolated PBMCs were stained as previously described (17, 18). Briefly, cells were stained with antibodies against CD33–fluorescein isothiocyanate (clone HIM3-4; BD Biosciences, Oxford, UK), HLA DR–phycoerythrin (clone G46-6; BD Biosciences), CD14–phycoerythrin–Cy7 (clone M5E2; BD Biosciences), and CD15–allophycocyanin (clone HI98; BioLegend, San Diego, CA, USA). 7-AAD viability dye (eBioscience, San Diego, CA, USA) was used to identify live cells. The cells were prepared for analyses and sorting as previously described (17, 18) Minimal sorter-induced cell stress was ensured following applicable measures, as previously described (19). FlowJo V10 software (FlowJo, Ashland, OR, USA) was used to perform data analyses.

### Library Preparation

Study design and pipeline for bioinformatics analyses were performed, as we have previously described (18). Briefly, 1,000 I-MDSCs, M-MDSCs, PMN-MDSCs, and monocytic APCs cells were sorted with high purity from PBMCs of CRC patients. The cDNA libraries were prepared from sorted cells using QIAseq FX Single Cell RNA Library Kit (Qiagen, Hilden, Germany), as we have previously reported (20). The quality passed libraries were subjected to sequencing using Illumina HiSeq 4000 as previously described (20).

### Transcriptomic Data Analyses

Raw data obtained from Illumina HiSeq 4000 in the form of FASTQ files were analyzed on CLC Genomics Workbench-12 (Qiagen), as previously described (18). Briefly, paired end reads were quality-trimmed and aligned to the hg19 human reference genome, and the read count was calculated as TPMs (transcripts per million) mapped reads. Default settings were applied to analyze the differential expression between the study subsets. The *Z* score was calculated for all the differentially expressed genes, with *P* < 0.05, and used for the construction of heat maps for data visualization.

### Functional Annotation Analyses

The Gene Ontology Biological Process (GO BP), Kyoto Encyclopedia of Genes and Genomes (KEGG), and BioCarta network analyses (21, 22) were performed on Database for Annotation, Visualization and Integrated Discovery platform (v.6.8, https://david.ncifcrf.gov/), as previously described (18). We focused on functional networks in upregulated/downregulated genes. The gene data from functional analyses were presented as heat plots. Protein–protein interaction networks for selected signaling pathways were obtained from STRING, a web-based online tool for protein–protein interaction networks and functional enrichment analyses (https://string-db.org/) (23). Principal component analysis (PCA) plots were generated using TPMs of differentially expressed genes on iDEP.91 (integrated Differential Expression and Pathway analysis, http://bioinformatics.sdstate.edu/idep/), a web-based online analyses tool, using default settings.

## Results

### Myeloid Subsets in the Circulation of CRC Patients

We have previously reported that levels of PMN-MDSCs were higher in the circulation of CRC patients, compared with healthy donors (3). Additionally, these PMN-MDSCs showed an upregulation of ARG1, which is an indicator of their suppressive function (3). Furthermore, we have previously reported that the levels of PMN-MDSCs and I-MDSCs are higher in the CRC TME (3). Using monocytic APCs as controls, our recent study on the transcriptomic characteristics of the different MDSC subsets in the TME of CRC patients has provided novel insights into the epigenetic mechanisms including DNA methylation/posttranslational histone modifications and other signaling pathways regulating their transcriptional profile and function (18). Despite the phenotypic differences between the various MDSC subsets, including granulocytic (PMN-), monocytic (M-), and immature (I-) MDSCs, they all possess an immunosuppressive activity and lack the expression of HLA-DR. On the contrary, monocytic APCs express HLA-DR; therefore, they were used as controls for the other three MDSC subsets.

**Figure 1:**
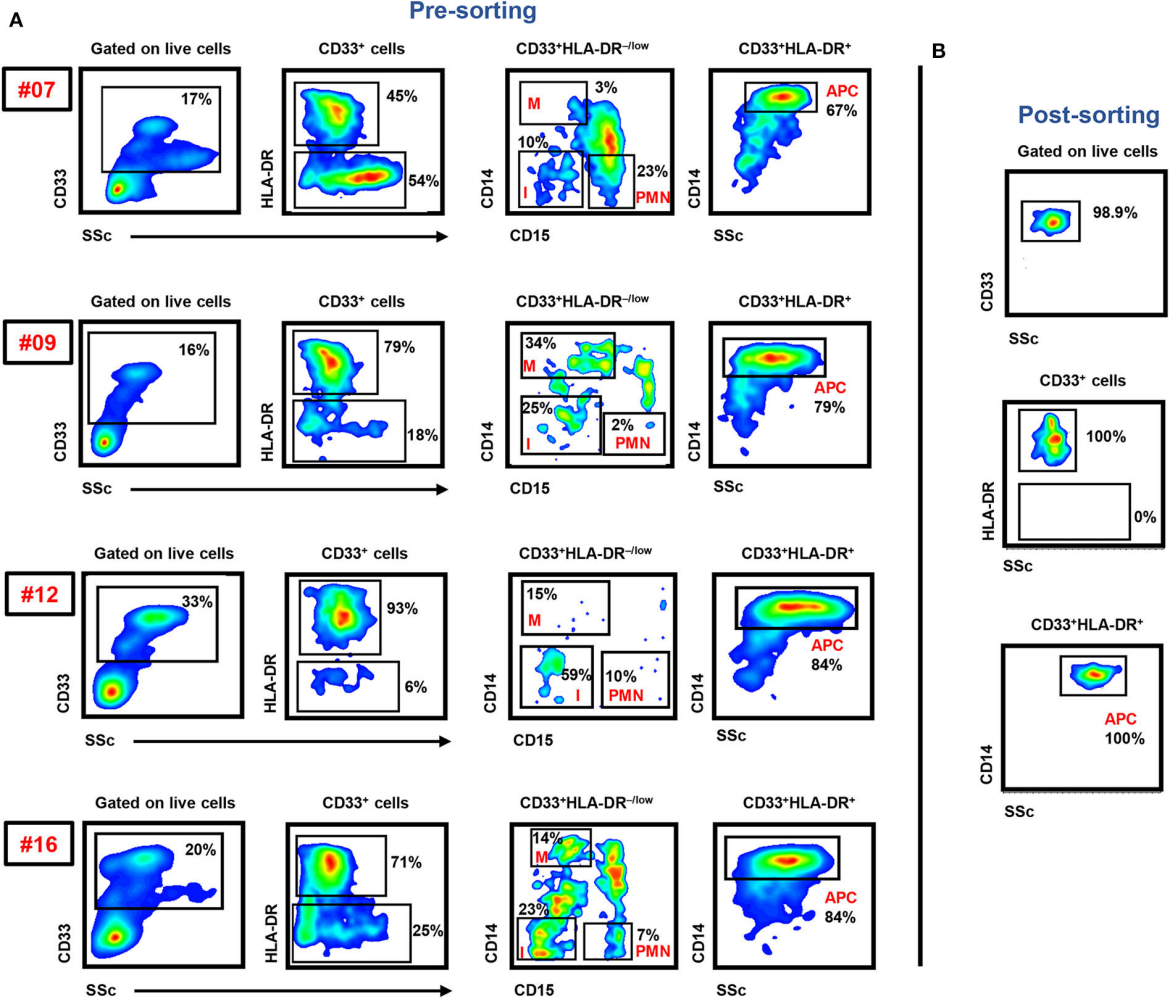
Gating strategy of I-MDSCs, M-MDSCs, PMN-MDSCs, and monocytic APCs. Peripheral blood mononuclear cells were isolated from four CRC patients (7, 9, 12, and 16). Flow cytometric plots show the levels of I-MDSCs (denoted as I), M-MDSCs (denoted as M), and PMN-MDSCs (denoted as PMN) gated on CD33^+^HLA-DR^−/low^ and monocytic APCs gated on CD33^+^HLA-DR^+^ cells. Sorted pure myeloid cell subsets were used for RNA-Seq **(A)**. Representative flow cytometric plots show the sorting purity of CD33^+^HLA-DR^+^CD14^+^ monocytic APCs **(B)**.

In this study, we sorted the different myeloid cell subpopulations including PMN-MDSCs (CD33^+^HLA-DR^−/low^CD14^−^CD15^+^), I-MDSCs (CD33^+^HLA-DR^−/low^CD14^−^CD15^−^), M-MDSCs (CD33^+^HLA-DR^−/low^CD14^+^CD15^−^), and monocytic APCs (CD33^+^HLA-DR^+^CD14^+^) from the circulation of CRC patients to investigate their transcriptomic characteristics, which could potentially contribute to disease progression. The gating strategy for sorting these subsets is shown in Figure 1. Comparative analyses were then performed from the libraries generated from the different sorted myeloid cell subpopulations, including PMN-MDSCs vs. monocytic APCs, PMN-MDSCs vs. I-MDSCs, M-MDSCs vs. monocytic APCs and I-MDSCs vs. monocytic APCs.

### Genes Associated With Janus Kinase–Signal Transducer and Activator of Transcription, Transcriptional Regulation, and Histone Acetylase Were Upregulated in Circulating PMN-MDSCs, Compared With Monocytic APCs of CRC Patients

**Figure 2:**
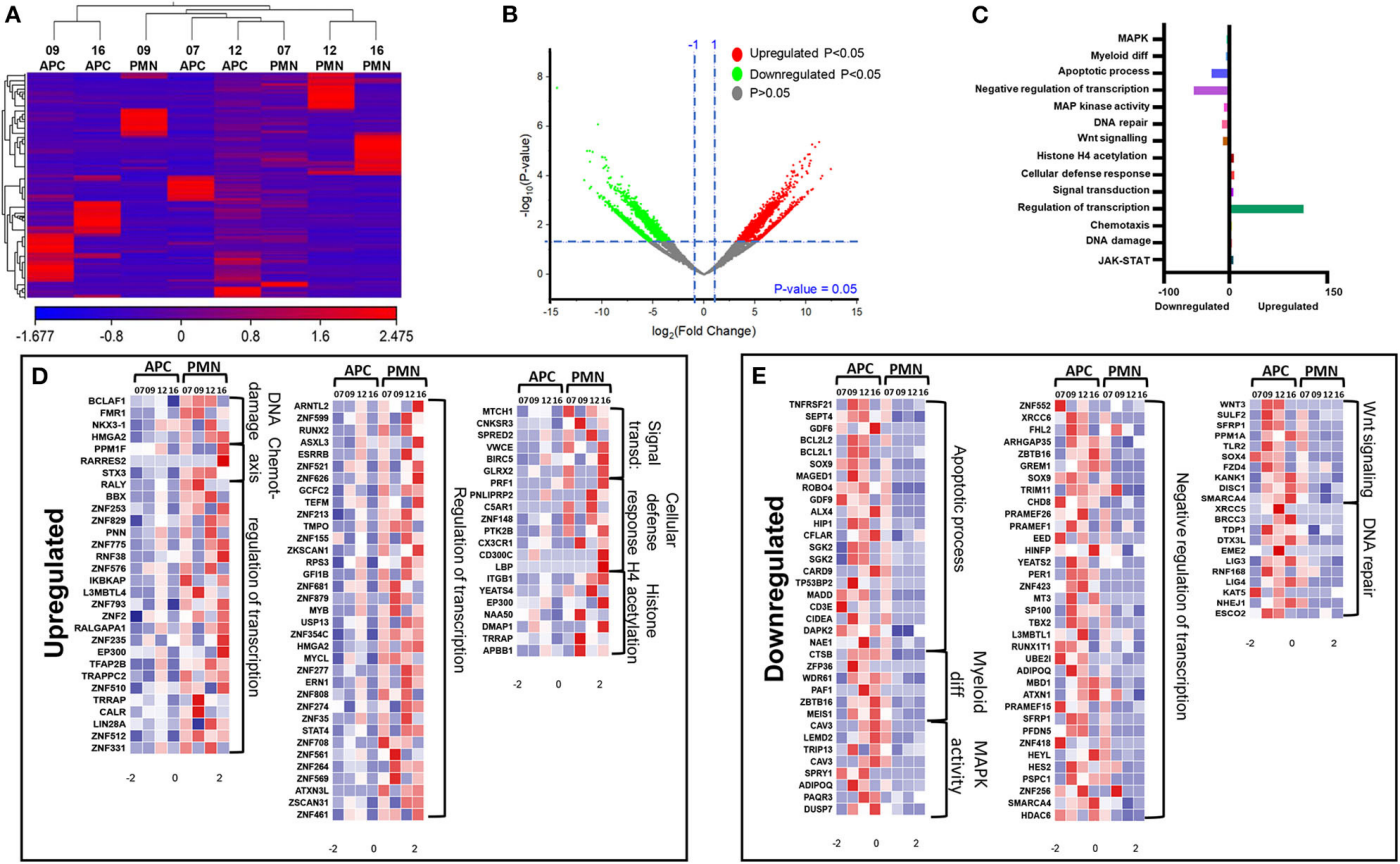
Comparative analyses of gene expression between PMN-MDSCs and monocytic APCs in the circulation of CRC patients. Hierarchical clustering of PMN-MDSCs and monocytic APCs from four patients (7, 9, 12, and 16) on differentially expressed RNA transcripts from RNA-Seq data. Each column represents a sample, and each row represents a transcript. The color gradient determines the expression level of each transcript **(A)**. Volcano plot shows differentially expressed genes; log2 fold change is plotted on the x-axis, and the statistical impact is shown on the y-axis. Fold changes with significant *P* values (>0.05) are highlighted in red (for upregulated genes) and green (for downregulated genes) **(B)**. Functional categorizations of significantly upregulated or downregulated genes were analyzed using DAVID platform. Bar diagram illustrates the percentage of genes in each functional category **(C)**. Heat maps show the *Z* score of upregulated **(D)** and downregulated **(E)** transcripts in PMN-MDSCs, compared with monocytic APCs. Color codes are shown; red indicates upregulation, white indicates no change, and blue indicates downregulation.

We have previously reported that DNA methylation- and histone deacetylase (HDAC) binding-related genes were downregulated in tumor-infiltrating PMN-MDSCs, compared with PMN-MDSCs in normal colon tissues of CRC patients (18). Here, we analyzed the transcriptomic profiles of PMN-MDSCs, compared with monocytic APCs in the circulation of four CRC patients (No. 7, 9, 12, and 16). The hierarchal clustering of differentially expressed transcripts showed 1,912 upregulated and 2,412 downregulated transcripts in PMN-MDSCs, compared with monocytic APCs (fold of change >2 and *P* value cutoff < 0.05) (Figures 2A,B). The PCA from the TPMs showed that the PMN-MDSCs and monocytic APCs from four patient samples were clustered separately, representing the significant differences in the overall gene expression (Supplementary Figure 1A). The PCA plot also showed high variability in the expression of PMN-MDSCs among the individuals, but it should not affect the downstream analyses due to the cluster separation of PMN-MDSCs and monocytic-APCs (Supplementary Figure 1A). Functional annotation analyses revealed that upregulated pathways in PMN-MDSCs were related to DNA damage (4 genes), chemotaxis (4 genes), transcriptional regulation (114 genes), signal transduction (6 genes), cellular defense response (8 genes), and Histone H4 acetylation (7 genes) (Figures 2C,D; Table 2). Interestingly, the genes responsible for apoptosis (21 genes), mitogen-activated protein kinase (MAPK) activity (8 genes), myeloid differentiation (6 genes), negative regulation of transcription (54 genes), and Wnt signaling pathway (10 genes) were downregulated in PMN-MDSCs (Figures 2C,E; Table 2). Moreover, genes involved in Wnt signaling pathway formed a PPI enrichment with *P* value 0.116, including 10 nodes and 4 edges (Supplementary Figure 2A).

**Table 2:** Summary of differentially regulated pathways in each group comparisons.

**Group comparison**	**Upregulated pathways**	**Downregulated pathways**
PMN-MDSCs vs. monocytic APCs	JAK-STAT signaling, DNA damage, chemotaxis, regulation of transcription, signal transduction, cellular defense response, histone H4 acetylation	Apoptotic process, myeloid cell differentiation, MAPK activity, negative regulation of transcription, Wnt signaling, DNA repair
PMN-MDSCs vs. I-MDSCs	DNA damage, methyl transferase activity, acetylation, apoptosis, DNA repair, MAPK signaling, Wnt signaling, and TGF-β signaling	Translational regulation, transcriptional regulation, IL-2, and IL-12 signaling
M-MDSCs vs. monocytic APCs	Transcription factor binding, PI3P binding, PI3K activation, protein dephosphorylation, regulation of transcription, apoptosis, histone H3 acetylation, T-cell proliferation, myeloid cell differentiation, IL-6 signaling, and TGF-β signaling	IFN-γ signaling, immune system regulation, and peptidyl-tyrosine dephosphorylation
I-MDSCs vs. monocytic APCs	Cell cycle and protein phosphorylation	Transcriptional coactivator activity, T-cell receptor signaling, positive regulation of transcription, and cell migration

**Figure 3:**
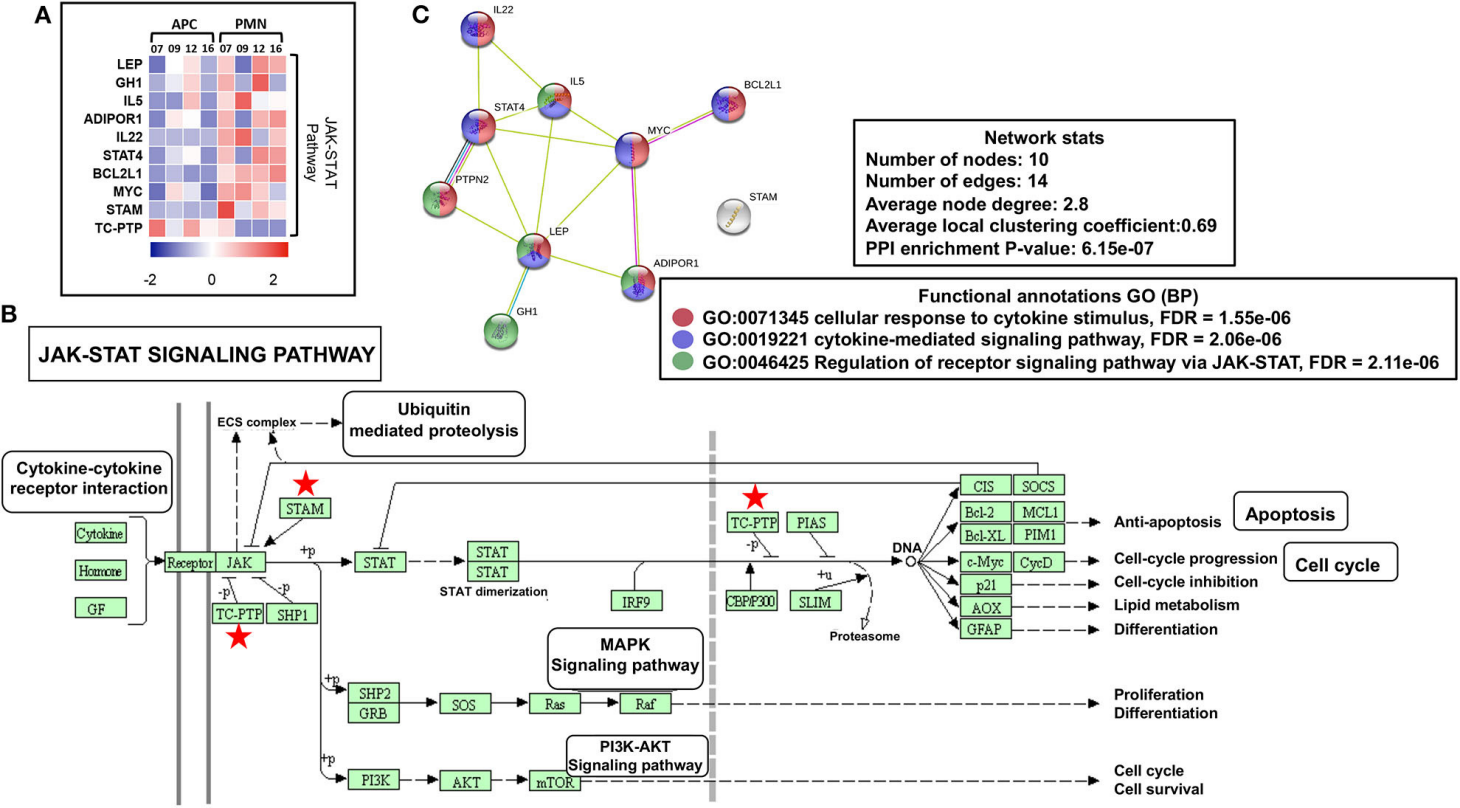
JAK-STAT modulating genes in PMN-MDSCs in the circulation of CRC patients. Heat map shows the *Z* score of JAK-STAT pathway-related transcripts that were downregulated/upregulated in PMN-MDSCs compared with monocytic APCs **(A)**. KEGG pathway analysis, using DAVID, showed genes (marked with red stars) involved in the JAK-STAT pathway **(B)**. PPI network analyses using the STRING database of genes from significantly deregulated genes related to JAK-STAT signaling pathway **(C)**. GO ontologies (color coded), description, and false discovery rate (FDR) using the whole transcriptome as reference are stated for each subnetwork. The overall network statistics are shown in the boxes.

Reports show that both leptin (LEP) (24) and growth hormone 1 (GH1) (25) could activate Janus kinase (JAK)–signal transducer and activator of transcription (STAT) cascade, which further induces angiogenesis, proliferation, and antiapoptotic pathways in normal cells and favors cancer progression. We found that both LEP and GH1 were upregulated in PMN-MDSCs, compared with monocytic APCs (Figure 3A). Furthermore, interleukin 5 (IL-5) and IL-22 were also upregulated in PMN-MDSCs, which are the potent activators of STAT-3 (26, 27). Protein tyrosine phosphatase nonreceptor type 2 (PTPN2 or TC-PTP), a known phosphorylation inhibitor of STAT1, STAT3, and STAT6 (28), was downregulated in PMN-MDSCs (Figures 3A,B). Moreover, IL-5/GH1, potent STAT3 activators, and STAT4 were upregulated in PMN-MDSCs (Figures 3A,B). In addition, the signal transducing adaptor molecule (*STAM*) gene, a known JAK signal accelerator (29), was upregulated in PMN-MDSCs (Figures 3A,B). Furthermore, PPI analysis showed that the vast majority of genes related to JAK-STAT pathway formed a network from 10 genes. STRING database identified 10 nodes and 14 edges with PPI enrichment *P* value 6.15e-07, average clustering coefficient of 0.69, and average node degree of 2.8 (Figure 3C). Collectively, these data suggest that the suppressive function and protumorigenic effects of circulating PMN-MDSCs in CRC could be associated with the upregulation of JAK-STAT pathway, activation of DNA damage cascade, and the downregulation of genes promoting tumor cell apoptosis and myeloid cell maturation. Moreover, our data imply that epigenetic modifications including histone acetylation could be involved in the transcriptional regulation of genes in circulating PMN-MDSCs of CRC patients.

### Genes Associated With Acetylation Were Upregulated in Circulating PMN-MDSCs, Compared With I-MDSCs

**Figure 4:**
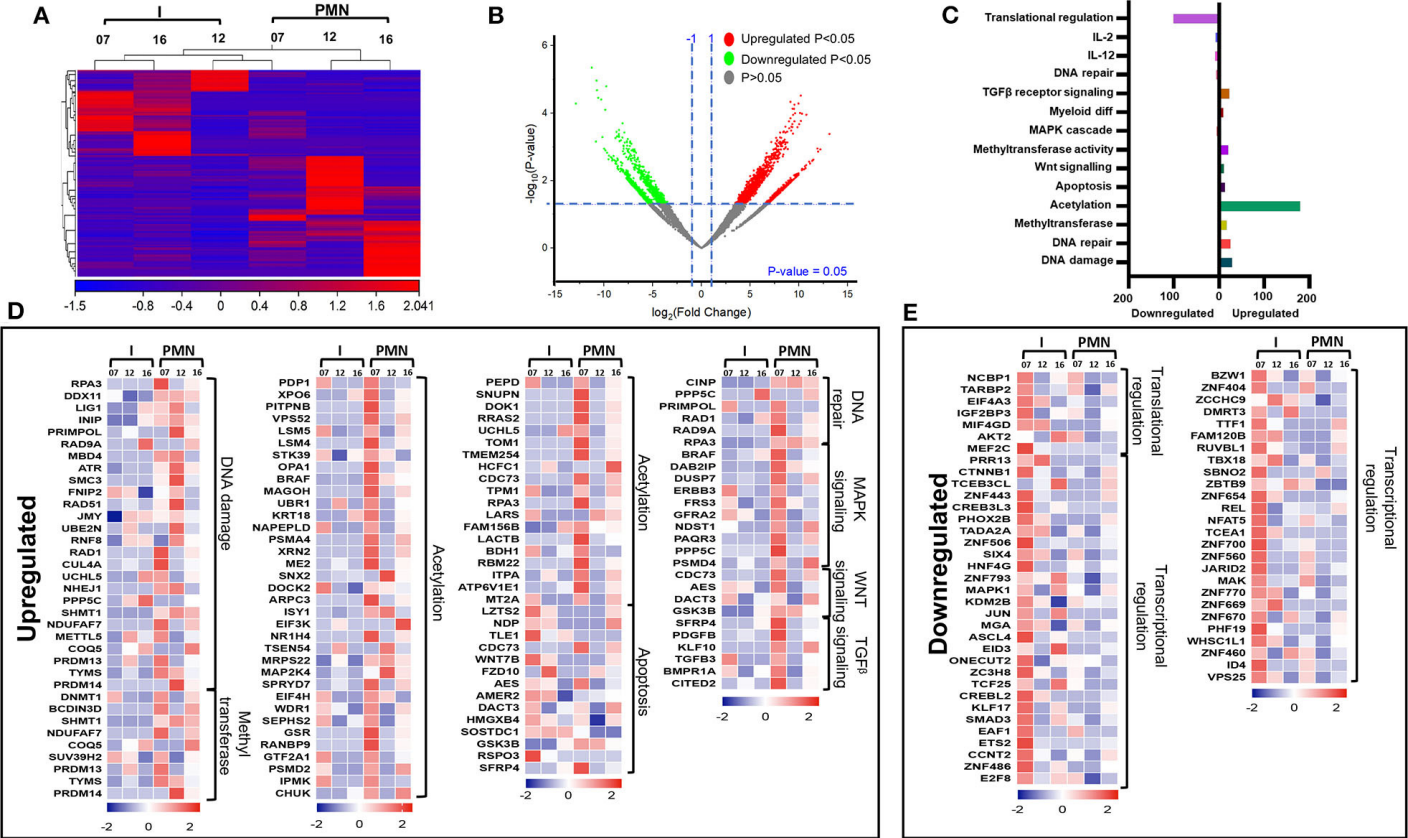
Comparative analyses of gene expression between PMN-MDSCs and I-MDSCs in the circulation of CRC patients. Hierarchical clustering of PMN-MDSCs and I-MDSCs from three patients (07, 12, and 16) on differentially expressed transcripts. Expression level of each gene is illustrated as a color code **(A)**. Volcano plot illustrates the differential expression data, represented as log2 fold change on the x-axis, with the statistical impact on the y-axis. Fold changes with significant *P* values (>0.05) are highlighted in red (for upregulated genes) and green (for downregulated genes) **(B)**. Functional categorizations for significantly upregulated and downregulated transcripts were analyzed on DAVID platform. Bar diagram illustrates the percentage of genes in each functional category **(C)**. Heat maps show the *Z* score of the upregulated **(D)** and downregulated **(E)** transcripts in PMN-MDSCs, compared with I-MDSCs, based on their functional categorization.

Next, we compared the transcriptomic profiles of PMN-MDSCs with I-MDSCs from three CRC patients (No. 7, 12, and 16). Sample 9 was not included in the analysis because of asymmetric clustering with other three samples. Hierarchical clustering of differentially expressed transcripts of PMN-MDSCs and I-MDSCs is shown in Figure 4A. There were 1,087 transcripts found to be upregulated and 861 downregulated in PMN-MDSCs, compared with I-MDSCs (FC >2 and *P* < 0.05) (Figures 4A,B). We found that 179 genes related to acetylation were upregulated in PMN-MDSCs, compared with I-MDSCs. Additionally, genes related to DNA damage (29 genes), apoptosis (14 genes), DNA repair (6 genes), MAPK signaling (11 genes), Wnt signaling (4 genes), and TGF-β signaling (6 genes) pathways were upregulated in PMN-MDSCs (Figures 4C,D; Table 2). It has been reported that TGF-β signaling pathway could activate MAPK signaling cascade and favors tumor progression (30). Interestingly, we found that TGF-β signaling–related genes including PDGFB, KLF10, and TGFB3 and MAPK-related genes including DUSP7, BRAF, CRBB3, and DAB2IP were significantly upregulated in PMN-MDSCs (Figure 4D). These data suggest that enhanced TGF-β signaling in PMN-MDSCs could activate MAPK signaling pathways for the survival and progression of CRC. On the other hand, 101 genes related to translational regulation were downregulated in PMN-MDSCs, compared with I-MDSCs (Figures 4C,E; Table 2). This latter finding suggests that genes involved in posttranslation modifications could be more important in PMN-MDSC function.

### Genes Associated With Apoptosis and Transcriptional Regulations Were Upregulated in Circulating M-MDSCs, Compared With Monocytic APCs of CRC Patients

**Figure 5:**
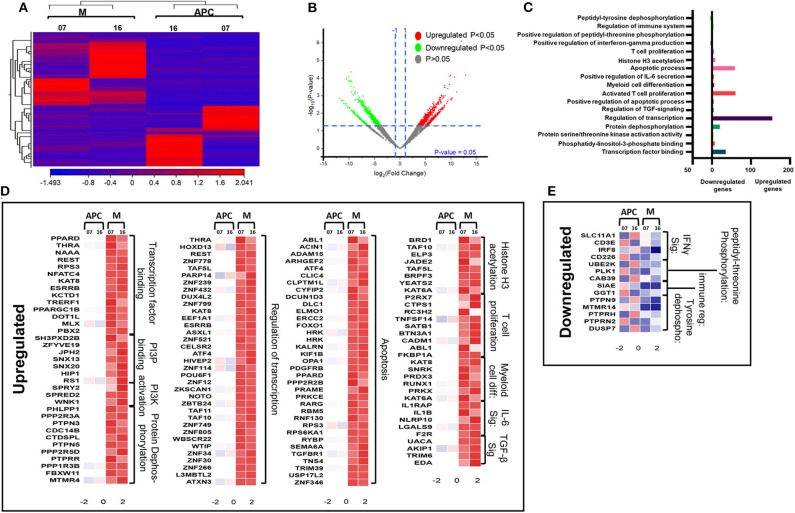
Comparative analyses of gene expression between M-MDSCs and monocytic APCs in the circulation of CRC patients. Hierarchical clustering of M-MDSCs and monocytic APCs from two patients (07 and 16) on differentially expressed transcripts. Gene expression level is illustrated as a color code **(A)**. Volcano plot illustrates the differential expression data, represented as log2 fold change on the x-axis, with the statistical impact on the y-axis. Fold changes with significant *P* values (>0.05) are highlighted in red (for upregulated genes) and green (for downregulated genes) **(B)**. Functional categorizations for significantly upregulated and downregulated transcripts were analyzed on DAVID platform. Bar diagram illustrates the percentage of genes in each functional category **(C)**. Heat maps show the *Z* score of the upregulated **(D)** and downregulated **(E)** transcripts in M-MDSCs, compared with monocytic APCs, based on their functional categorization.

Next, we compared the transcriptomic profiles of circulating M-MDSCs and monocytic APCs from two CRC patients (No. 7 and 16). The hierarchal clustering of differentially expressed transcripts of M-MDSCs and monocytic APCs is shown in Figure 5A. There were 1,719 transcripts found to be upregulated and 371 downregulated in M-MDSCs, compared with those found in monocytic APCs (FC >2 and *P* < 0.05) (Figures 5A,B). PCAs of the total data sets confirmed the close relativeness of biological replicates (Supplementary Figure 1B). Functional annotation analyses showed that transcription factor binding (35 genes)–, PI3P (7 genes)–, PI3K activation (3 genes)–, protein dephosphorylation (20 genes)–, transcriptional regulation (155 genes)–, apoptosis (59 genes)–, histone H3 acetylation (8 genes)–, and IL-6 pathway (5 genes)–related genes were upregulated in M-MDSCs, compared with monocytic APCs (Figures 5C,D; Table 2). Additionally, our PPI network analyses showed that there is a significant enrichment of genes related to IL-6 pathway including five nodes and three edges with PPI enrichment *P* = 0.000605 (Supplementary Figure 2B). Notably, genes related to interferon γ (IFN-γ) (SLC11A1, CD3E, IRF8, and CD226) and immune regulation (SIAE and GGT1) were downregulated in M-MDSCs (Figures 5C,E). It has been reported that STAT1/IFN-γ signaling pathway was regulated by a novel tumor suppressor protein, IRF8 (31). Moreover, downregulation of IRF8 was evident in breast tumor conditions leading to the progression of disease (31). In agreement with this, we found that IRF8 was significantly downregulated in M-MDSCs, which could be the potent rationale for the downregulation of immune regulation–related genes (Figure 5E). These results suggest that circulating M-MDSCs could contribute to CRC progression by suppressing the gene expression of immune-regulatory molecules involved in the activation of immune responses and upregulating genes involved in histone acetylation and transcriptional regulation.

### Genes Associated With Cell Migration and Transcriptional Regulations Were Downregulated in Circulating I-MDSCs, Compared With Monocytic APCs of CRC Patients

**Figure 6:**
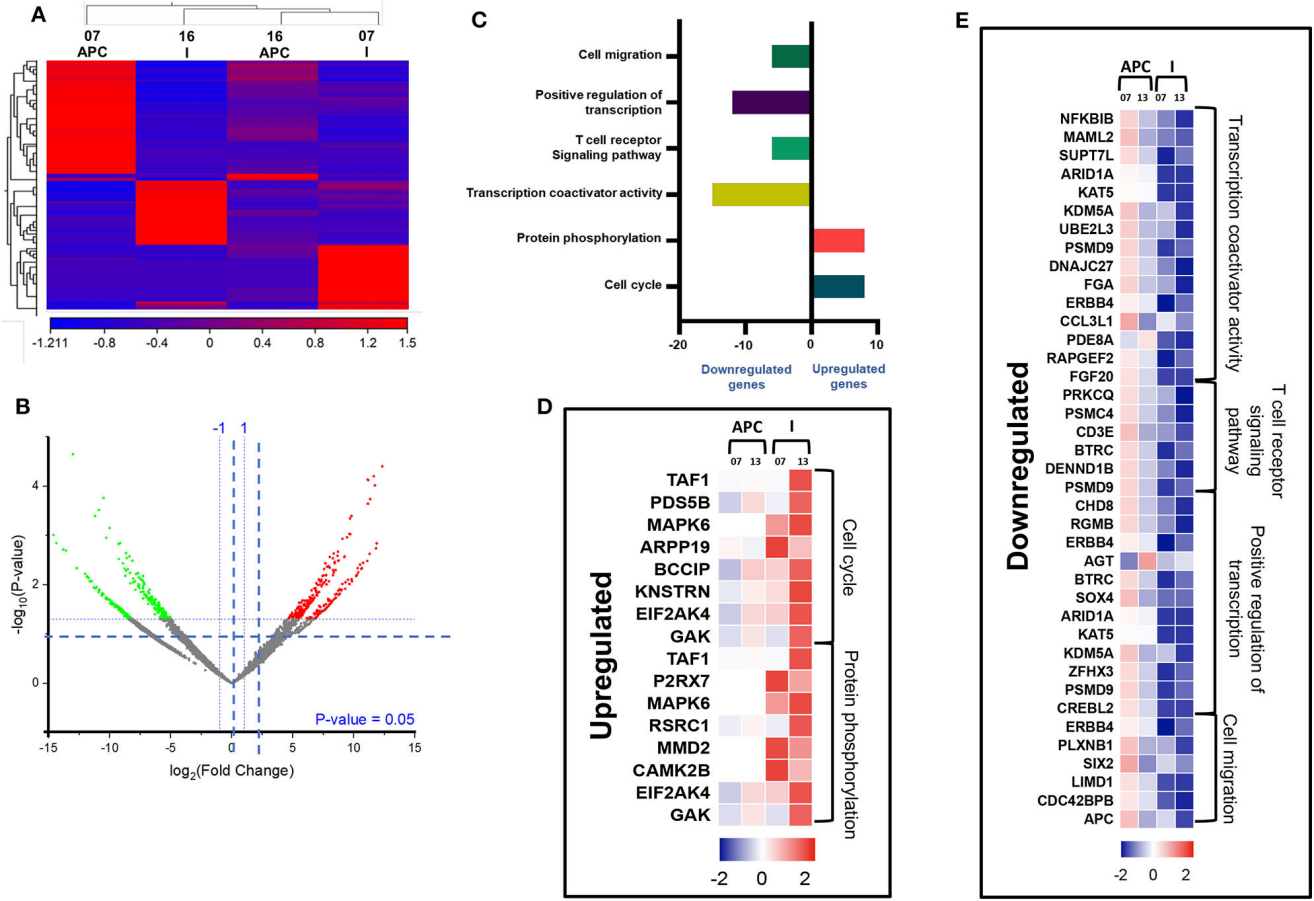
Comparative analyses of gene expression between I-MDSCs and monocytic APCs in the circulation of CRC patients. Hierarchical clustering of I-MDSCs and monocytic APCs from two patients (07 and 16) on differentially expressed transcripts. Expression level of each gene is illustrated as a color code **(A)**. Volcano plot illustrates the differential expression data, represented as log2 fold change on the x-axis, with the statistical impact on the y-axis. Fold changes with significant *P* values (>0.05) are highlighted in red (for upregulated genes) and green (for downregulated genes) **(B)**. Functional categorizations for significantly upregulated and downregulated transcripts were analyzed on DAVID platform. Bar diagram illustrates the percentage of genes in each functional category **(C)**. Heat maps show *Z* score of the upregulated **(D)** and downregulated **(E)** transcripts in I-MDSCs, compared with monocytic APCs, based on their functional categorization.

We then compared the transcriptomic profiles of I-MDSCs with monocytic APCs from two CRC patients (No. 7 and 16). The differentially expressed transcripts are shown as distinct cluster of I-MDSCs and monocytic APCs (Figure 6A). One hundred twenty-nine transcripts were found to be upregulated, and 275 were downregulated in I-MDSCs, compared with those found in monocytic APCs (FC >2 and *P* < 0.05) (Figures 6A,B). PCAs of the total data sets confirmed the close relativeness of biological replicates (Supplementary Figure 1C). Functional annotation analyses showed that cell cycle (8 genes)– and protein phosphorylation (8 genes)–related genes were upregulated (Figures 6C,D), and transcriptional (30 genes)– and cell migration (6 genes)–related genes were downregulated in I-MDSCs (Figures 6C,E; Table 2).

### Genes Associated With Acetylation Are Upregulated in PMN-MDSCs and M-MDSCs in the Circulation of CRC Patients

**Figure 7:**
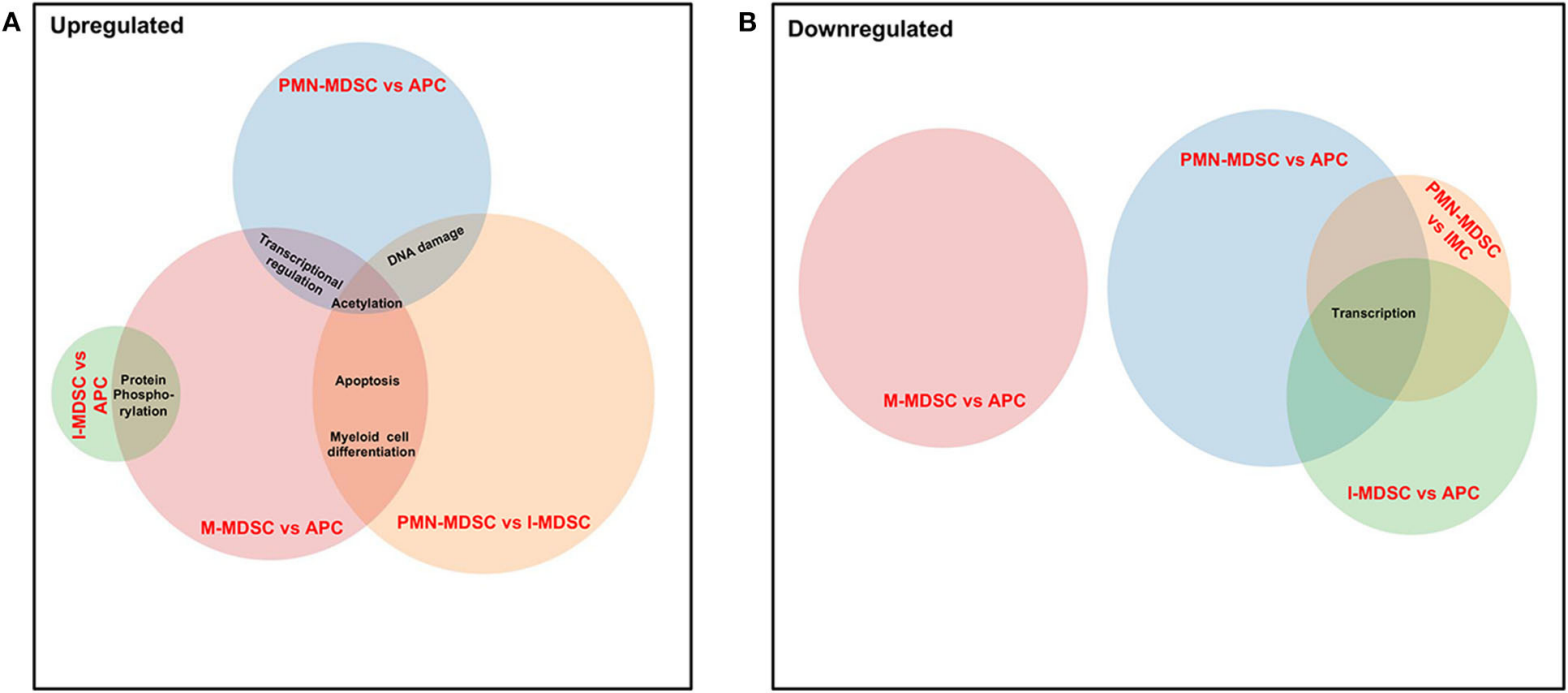
Analyses of overlapping functional pathways between PMN-MDSCs, I-MDSCs, and M-MDSCs. Venn diagram summarizing the overlap between functional pathways, which were upregulated **(A)** and downregulated **(B)** in the comparative analyses between PMN-MDSCs vs. monocytic APCs, PMN-MDSCs vs. I-MDSCs, M-MDSCs vs. monocytic APCs, and I-MDSCs vs. monocytic APCs. Shared pathways are indicated by the overlap between circles.

Finally, we compared the common pathways, which were upregulated/downregulated in PMN-MDSCs, M-MDSCs, and I-MDSCs in the circulation of CRC patients. We found that by comparing PMN-MDSCs vs. monocytic APCs, M-MDSCs vs. monocytic APCs, and PMN-MDSCs vs. I-MDSCs, genes related to acetylation were upregulated in PMN-MDSCs and M-MDSCs (Figure 7A), and also genes related to apoptosis and myeloid cell differentiation were upregulated in PMN-MDSCs and M-MDSCs (Figure 7A). Moreover, transcriptional regulation–related genes were upregulated in PMN-MDSCs and M-MDSCs; when comparing PMN-MDSCs vs. monocytic APCs and M-MDSCs vs. monocytic APCs (Figure 7A), DNA damage–related genes were upregulated in PMN-MDSCs, compared to monocytic APCs and I-MDSCs (Figure 7A). On the other hand, protein phosphorylation–related genes were upregulated in both I-MDSCs and M-MDSCs, compared to their controls (Figure 7A). In the downregulated pathways panel, genes related to transcription were downregulated in PMN-MDSCs and I-MDSCs, when comparing PMN-MDSCs vs. monocytic APCs, I-MDSCs vs. monocytic APCs, and PMN-MDSCs vs. I-MDSCs (Figure 7B).

## Discussion

In this study, we performed comparative analyses on transcriptomic profiles of different myeloid cell subsets in the circulation of CRC patients. We found that various signaling pathways, including JAK-STAT, chemotaxis, and histone acetylation, were upregulated in PMN-MDSCs compared with monocytic APC: PI3P kinase, PI3 kinase, apoptosis, H3 acetylation, IL-6, and TGF-β pathways were upregulated in M-MDSCs, and cell cycle–related pathways were upregulated in I-MDSCs compared with monocytic APCs.

Tumor-infiltrating inflammatory cells are reported to be associated with disease progression; however, the functional impact of particular subpopulations remains not evident (32). Recently, we have reported that in the CRC TME, levels of PMN-MDSCs, and I-MDSCs were higher compared with M-MDSCs (18). Other than CRC, in lung cancer patients, it has been reported that PMN-MDSCs accumulated in the advanced stages have predominant suppressive characteristics and favor tumor progression (33). We have previously shown that various epigenetic-related genes including DNA methylation and HDACs were upregulated, and acetylation-related genes were downregulated in colorectal tumor-infiltrating I-MDSCs, compared with monocytic APCs (18). Notably, in the present study, we found that acetylation-related genes were upregulated in PMN-MDSCs, compared with monocytic APCs, in circulation of CRC patients. Furthermore, in the CRC TME, DNA methylation and HDACs were downregulated in PMN-MDSCs, compared with monocytic APCs (18). In line with these data, we found that histone acetylation– and transcription regulation–related genes were upregulated in circulating PMN-MDSCs. These data show that transcriptional regulation of pivotal genes in circulating PMN-MDSCs might be regulated by posttranslational histone modifications. Moreover, our work showed that MAPK signaling and Wnt pathways were upregulated in PMN-MDSCs of both TME (18) and circulation, compared with I-MDSCs.

Tumor-infiltrating immune cells, including myeloid cells, provide an immune-subversive environment for tumor development and progression through the activation of various signaling cascades (34). It has been reported that the MDSC subset, I-MDSCs, favors tumor progression by expanding T_H_17 cells within the TME, rather than suppressing T cells (35). We found that critical pathways for tumor progression associated with DNA damage, MAPK, and Wnt signaling were upregulated in PMN-MDSCs, compared with I-MDSCs. Interestingly, JAK-STAT pathway was also found to be upregulated in PMN-MDSCs, compared with monocytic APCs. JAK-STAT pathway is one of the predominant signaling cascades, which can promote immune suppression and tumor cell survival (36). JAK-STAT signaling cascade has been implicated in the induction of chemotaxis, which in turn triggers migratory signals in epithelial cells causing cell transformation from being static epithelial cells to migratory cells (37). Interestingly, we found that STAT3- and STAT4-related genes were upregulated in circulating PMN-MDSCs, compared with monocytic APCs. Reports showed that growth hormone induces the activation of Hrs–STAM complex, which could enhance the formation of JAK–receptor complexes (29, 38). In agreement with these reports, we found that GH1 and STAM were upregulated in PMN-MDSCs, compared with monocytic APCs. Aberrant activation of JAK-STAT signaling pathway could also activate MAPK pathway, which controls various fundamental cellular processes that promote tumorigenesis, including cell proliferation, survival, differentiation, and migration (39, 40). Furthermore, components of the JAK-STAT signaling pathway and its downstream cascade MAPK pathway could induce regulatory T cell (Treg) and MDSC expansion and interfere with T_H_1 differentiation (41, 42). Collectively, these data imply that JAK-STAT signaling pathway in circulating PMN-MDSCs could be important for triggering their own proliferation and Treg expansion, contributing to malignant cell migration and metastasis, and possibly opposing T_H_1 differentiation and migration into the TME of CRC patients (43).

One of the most frequently altered pathways in human cancer is the PI3K pathway, which has a critical role in tumorigenesis and tumor progression (44). Here, we found that PI3K and PI3P pathways were upregulated in M-MDSCs, compared with monocytic APCs, which could activate multiple downstream pathways and favor malignant cell progression. It has been reported that the activation of PI3K could trigger PI3P signaling cascade and promote the survival of tumor cells (45). Additionally, genes related to IL-6 and TGF-β signaling pathways were found to be upregulated in M-MDSCs, compared to monocytic APCs. Overexpression of TGF-β has been reported to promote tumorigenesis as it induces multiple non-Smad pathways, which could reprogram epithelial cells and induce epithelial–mesenchymal transition (46). This overexpression of TGF-β was correlated with advanced disease stages, metastasis, and poor prognosis in many cancer types, including CRC (46–49). Moreover, TGF-β released by MDSCs can stimulate Treg expansion and survival and enhance their suppressive activity (50, 51). In turn, Tregs can trigger the induction of MDSCs, thereby creating a positive feedback loop, favoring immune suppression and T effector cell (Teff) apoptosis or inactivation (50, 52). Reports showed that several non-Smad signaling pathways could be activated by TGF-β, such as PI3K/AKT and IL-6 signaling, leading to resistance to various cancer treatments (47, 53, 54). These data suggest that M-MDSCs in the circulation of CRC patients upregulate multiple pathways, including TGF-β, IL-6, PI3P, and PI3K, which could promote tumorigenesis by supporting Treg expansion and suppressive activity and favoring immune suppression and therapy resistance.

Signaling cascades are regulated by protein kinases, which are activated or deactivated through phosphorylation and dephosphorylation. Here, we found that, in I-MDSCs, both cell cycle– and protein phosphorylation–related genes were upregulated, compared with monocytic APCs. These genes could be associated with the activation of signaling cascades, which govern cellular processes, such as cell proliferation, migration, differentiation, survival, and growth of immune-suppressive cells, and regulating Teff function and migration into the TME (55).

Epigenetic modifications via DNA methylation and histone methylation/acetylation have also a major impact on tumorigenesis, immunosuppression, and cancer progression (18, 56, 57). Additionally, acetylation and deacetylation of both histone and nonhistone marks have been reported to be closely associated with the transcriptomic regulation of genes associated with cell proliferation, cell cycle, and apoptosis in cancer. Here, we found that acetylation-related genes were upregulated in PMN-MDSCs and M-MDSCs, compared with monocytic APCs. Apart from acetylation, genes related to transcription, DNA damage, apoptosis, and myeloid differentiation were also found to be upregulated in PMN-MDSCs and M-MDSCs. These data suggest that elevated expression of acetylation-related genes in MDSCs could trigger multiple signaling cascades promoting the survival and progression of malignant cells. However, the suppressive function of these cells in human is not totally evident.

Some of the signaling pathways investigated in this study were exclusively upregulated in one myeloid suppressive subset, implicating the importance of their functions and contribution to CRC progression. Additionally, this study revealed some of the epigenetic mechanisms, which control the transcriptional profile of the different myeloid cell subsets. However, further studies are required to validate these findings using functional assays and in larger patient cohorts. Additionally, it would be interesting to compare the transcriptomic profiles of circulating MSDC subsets between CRC patients and healthy donors to identify distinct enrichment of transcripts/pathways in specific subsets favoring onset and progression of CRC.

## Data Availability Statement

The datasets presented in this study can be found in the following online repository: Sequence Read Archive https://www.ncbi.nlm.nih.gov/sra/?term=PRJNA641527.

## Ethics Statement

The studies involving human participants were reviewed and approved by Institutional Review Board, Hamad Medical Corporation, Doha, Qatar; and Institutional Review Board, Qatar Biomedical Research Institute, Hamad Bin Khalifa University, Qatar Foundation, Doha, Qatar.

## Author Contributions

VS performed experimental work, data analyses and wrote the manuscript. RS and ST assisted in experimental work. NA performed bioinformatics and data analysis and reviewed the manuscript. EE conceived the idea, designed the study, supervised the project, analyzed and interpreted data, and wrote and revised the manuscript. All authors were involved in the final approval of the manuscript.

## Conflict of Interest

The authors declare that the research was conducted in the absence of any commercial or financial relationships that could be construed as a potential conflict of interest.
