# Supplementary material for: Transcriptomic Analyses of Myeloid-Derived Suppressor Cell Subsets in the Circulation of Colorectal Cancer Patients
Source: Front Oncol. 2020 Sep 2;10:1530. doi: 10.3389/fonc.2020.01530 (PMC7492613; doi:10.3389/fonc.2020.01530)
Supplement: Supplementary file 1 [file Data_Sheet_1.PDF]

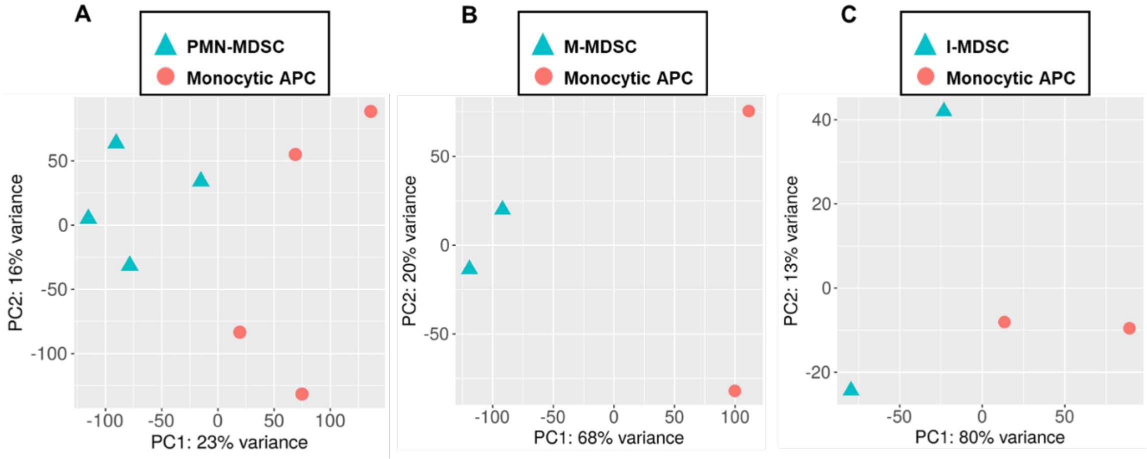

Supplementary Figure 1. Principal component analysis (PCA) on gene expression data. The PCA plots show the percentage of variation between the differentially expressed genes from PMN-MDSC vs. monocytic APC (A), M-MDSC vs. monocytic APC (B) and I-MDSC vs. monocytic APC (C). Each data point represents a single sample.

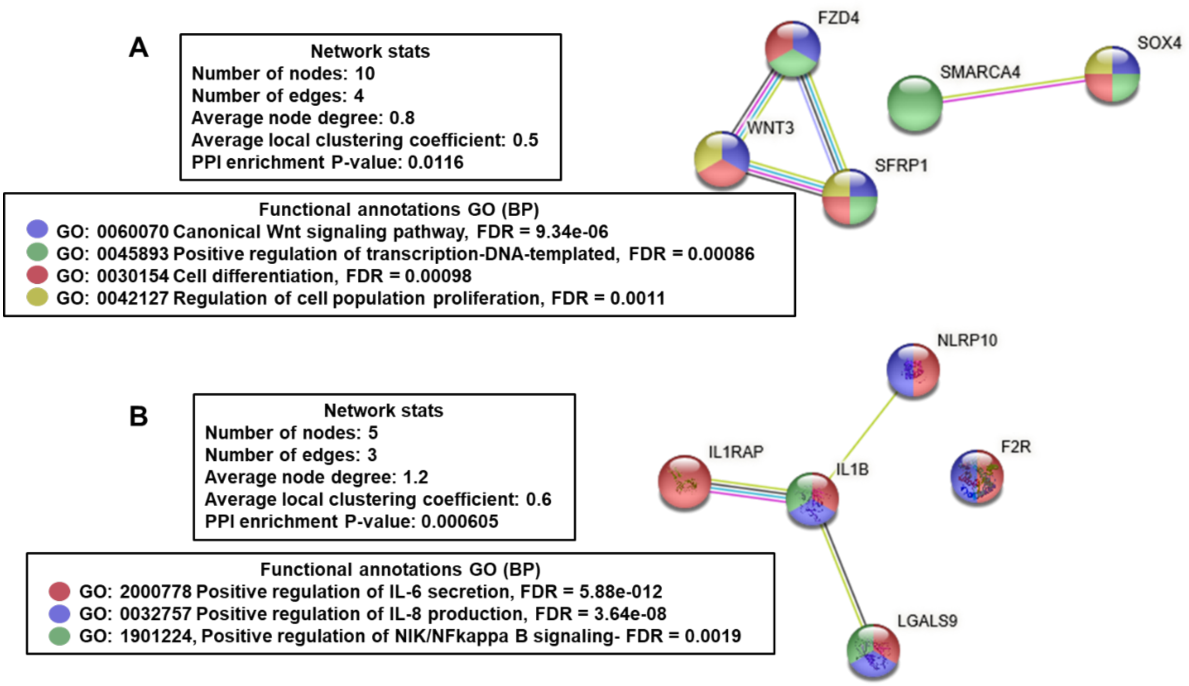

Supplementary Figure 2. Protein-protein interaction (PPI) network analyses of deregulated genes from PMN-MDSC and M-MDSC. PPI network analyses using the STRING database of genes from Wnt signaling pathway; downregulated in PMN-MDSC (A) and IL-6 signaling pathway; upregulated in M-MDSC (B), compared with monocytic APCs. GO ontologies (color coded), description and false discovery rate (FDR) using the whole transcriptome as reference are stated for each subnetwork. The overall network statistics are shown in the boxes.
